# Epidemiological Data Mining for Assisting with Foodborne Outbreak Investigation

**DOI:** 10.3390/foods12203825

**Published:** 2023-10-19

**Authors:** Dandan Tao, Dongyu Zhang, Ruofan Hu, Elke Rundensteiner, Hao Feng

**Affiliations:** 1 Vanke School of Public Health, Tsinghua University, Beijing 100084, China; dandantao@mail.tsinghua.edu.cn; 2 Data Science Program, Worcester Polytechnic Institute, Worcester, MA 01609, USA; dzhang5@wpi.edu (D.Z.); rhu@wpi.edu (R.H.); 3 Department of Computer Science, Worcester Polytechnic Institute, Worcester, MA 01609, USA; 4 College of Agriculture & Environmental Sciences, North Carolina A & T State University, Greensboro, NC 27411, USA

**Keywords:** foodborne diseases, outbreak investigation, epidemiological data, data mining, network analysis, cross contamination

## Abstract

Diseases caused by the consumption of food are a significant but avoidable public health issue, and identifying the source of contamination is a key step in an outbreak investigation to prevent foodborne illnesses. Historical foodborne outbreaks provide rich data on critical attributes such as outbreak factors, food vehicles, and etiologies, and an improved understanding of the relationships between these attributes could provide insights for developing effective food safety interventions. The purpose of this study was to identify hidden patterns underlying the relations between the critical attributes involved in historical foodborne outbreaks through data mining approaches. A statistical analysis was used to identify the associations between outbreak factors and food sources, and the factors that were strongly significant were selected as predictive factors for food vehicles. A multinomial prediction model was built based on factors selected for predicting “simple” foods (beef, dairy, and vegetables) as sources of outbreaks. In addition, the relations between the food vehicles and common etiologies were investigated through text mining approaches (support vector machines, logistic regression, random forest, and naïve Bayes). A support vector machine model was identified as the optimal model to predict etiologies from the occurrence of food vehicles. Association rules also indicated the specific food vehicles that have strong relations to the etiologies. Meanwhile, a food ingredient network describing the relationships between foods and ingredients was constructed and used with Monte Carlo simulation to predict possible ingredients from foods that cause an outbreak. The simulated results were confirmed with foods and ingredients that are already known to cause historical foodborne outbreaks. The method could provide insights into the prediction of the possible ingredient sources of contamination when given the name of a food. The results could provide insights into the early identification of food sources of contamination and assist in future outbreak investigations. The data-driven approach will provide a new perspective and strategies for discovering hidden knowledge from massive data.

## 1. Introduction

Foodborne disease is a major public health challenge. Despite advances in food safety regulation, prevention technology, and education, foodborne illness persists, and foodborne outbreaks continue to occur [1]. In the United States, the Centers for Disease Control and Prevention (CDC) estimated that each year, roughly 48 million illnesses, 128,000 hospitalizations, and 3000 deaths are attributed to the consumption of contaminated foods [2]. A foodborne outbreak was defined by the CDC as an event when two or more persons experience a similar illness due to the consumption of a common food [3]. Identifying the source of contamination is a key step in an outbreak investigation to prevent foodborne illnesses. Source attribution, defined as attributing a foodborne disease to a specific food or commodity, is regarded as an important process in food safety risk management for enabling effective control measures to improve food safety and help prevent future diseases [4]. There are several methods that are commonly used in source attribution, including the microbial subtyping approach, comparative exposure assessment, expert elicitation, the epidemiological analysis of sporadic cases, and the analysis of data from outbreak investigations [5]. The last two methods rely on epidemiological principles.

Epidemiological data analysis was proven to be an effective method for assisting with outbreak investigation by identifying potential risks to human health [6]. Historical foodborne outbreaks provide critical information on the factors contributing to specific food contamination and the foods causing the illnesses. An analysis of outbreaks across several years can provide food safety managers with ways to monitor trends over time for outbreaks caused by specific etiologies and foods, which will provide valuable information about future outbreak prevention [7]. Considerable work has been conducted to analyze foodborne outbreak data based on the pathogen, food, or the food/pathogen combination through descriptive approaches [4,8,9,10]. The expert elicitation method was used to link foodborne illnesses caused by different pathogens to the consumption of different types of food [8]. The association between food vehicles and etiologies was estimated across multiple countries through the analysis of foodborne outbreaks [4]. In recent years, data-driven approaches such as data mining and machine learning have been employed to analyze foodborne outbreaks for predictive purposes. For example, decision tree and association rules were applied to predict specific etiologies regarding food vehicles and locations [11]. More recently, machine learning methods such as supervised learning have been used to identify patterns in foodborne illnesses from online big data in comparison with traditional outbreak data [12,13]. In addition, food source attribution is significant in outbreak investigation to mitigate the spreading of foodborne diseases. Hence, national or international databases with records of historical foodborne outbreaks have been widely used to estimate the contribution of different food sources to outbreaks caused by different etiologies [4,14,15].

The food system is becoming more complex with the rising trends of globalization and industrialization, which necessitates the identification of relations in the critical attributes of persistent foodborne outbreaks for the prevention of future outbreaks. Therefore, this work aims to identify (1) the relations between contributing factors and food vehicles, (2) the relations between food vehicles and etiologies through data mining approaches, and (3) understanding the role of simple/complex foods and ingredients in different outbreaks and the relations between them. The methodologies provide new approaches to analyze massive data and non-structured data such as text data to assist with outbreak investigation in an automatic way. The discovered knowledge will be useful for designing food safety intervention strategies in outbreak prevention.

## 2. Related Work

Most of the reported illnesses are sporadic and are not associated with an outbreak. Typically, local health departments report serious cases of foodborne illnesses to the CDC. The reported data will be investigated by the CDC to obtain information regarding the food that likely played a role in the outbreak. Determining the etiology, food vehicles, and the contributing factors is the critical task for designing control measures in outbreak investigation. Specifically, the identification of the food vehicle and the etiology causing the foodborne outbreak is significant in the prevention of additional illnesses [4]. The sooner they can identify the food source of contamination, the sooner they can implement control measures such as the recall of the food to prevent the spread of illness. While statistical methods have traditionally been used in historical foodborne outbreak investigations, advanced computational methods such as data mining, network analysis, and the Monte Carlo simulation have provided new perspectives and insights to assist the process in multiple stages. With the globalization of food production and distribution, the potential for foodborne illnesses and outbreaks has grown significantly. Recently, the field of data mining has emerged as a powerful tool in the realm of food safety. Data mining, a sub-set of the broader field of data analytics, involves the extraction of valuable insights and patterns from large datasets [16]. For example, a typical data mining technique, association rules, was used to identify the relations between foods, places, and etiologies involved in historical outbreaks to provide insights into good food safety practices [11].

A network analysis is a relation analysis method used to analyze graph-structured data. It is the science of understanding the connectivity of items in specific systems and what the connections do. Networks of food-related words can be constructed from the association of word pairs. The past decade has seen applications of the network analysis of digital text data to improve our understanding of food science and nutrition. For example, a flavor network was constructed based on online recipes and was analyzed to provide ideas of creative cooking, in which the node represented a name of an ingredient, and the edge represented the shared flavor compounds between two ingredients [17,18]. In another application, the networks of diseases and food nutrients were built based on text mining of the literature, in which the node represented the name of a disease or food nutrient, and the edge represented the evidence of the food nutrient in treating the disease [19,20,21]. By defining different meanings for nodes and edges, different networks can be constructed to describe the relations between items. Based on the constructed network, a variety of network analyses can be implemented. For example, centrality measures are often employed to identify the most important nodes in a network [22]. Centrality measures include degree centrality, betweenness centrality, closeness centrality, and eigenvector centrality depending on different definitions of importance.

A Monte Carlo simulation is a numerical technique typically used in qualitative risk assessment to address microbial food safety problems [23]. Through the random selection of input values based on the probability distributions and iterations for several thousand times, a Monte Carlo analysis produces different results on different runs. The output of the analysis is a frequency distribution representing the combined ranges and frequencies of the inputs [24]. In addition to food safety applications, Monte Carlo simulation has been used in other food-related topics such as testing sample pooling strategies [25], modeling light transport in food and agricultural products [26], associating environmental footprints with meat production and consumption [27], and estimating uncertainty intervals for nutrient values in food products [28].

## 3. Materials and Methods

### 3.1. Data Source

National data on reported foodborne outbreaks are available from the Centers for Disease and Prevention’s (CDC) Foodborne Outbreak Surveillance System, i.e., the National Outbreak Reporting System (NORS, https://www.cdc.gov/nors/index.html, accessed on 7 December 2018), which is a sub-set of the Electronic Foodborne Outbreak Surveillance System (eFORS). Data on foodborne and waterborne enteric disease outbreaks from 1998 to 2008 were collected using the eFORS. Since 2009, state, local, and territorial public health departments have electronically reported the data to NORS in place of eFORS, and the new system was expanded to collect data on foodborne, waterborne, person-to-person, animal contact, environmental contamination, and undetermined transmission routes (Hall et al., 2014) [29]. In this study, foodborne outbreaks reported to the CDC from 1998 to 2017 were extracted from the NORS using the following qualifications: mode of transmission of food, finalized outbreak report, onset year between 1998 and 2017, and number of estimated primary illnesses greater than 1. In addition, demographic data related to the outbreaks were provided by the CDC with a Microsoft Access database, in which the relational data were merged into a single, flat file using Python 3.7.0. Basic statistics of the NORS data can be seen in Figure S2. Ingredient dataset was downloaded from the USDA foodData Central, which includes the National Health and Nutrition Examination Survey (NHANES) for characterizing what people eat in the United States. NHANES was used to obtain standardized and reproducible sources of data about the ingredients of common foods consumed in the United States.

### 3.2. Experiment Design

We will first explore the raw dataset for two questions: (1) For outbreaks caused by “simple” food (a single contaminated ingredient or a food containing multiple ingredients but belonging to a single food category), what are the predictable contributing factors (X: $X_{1}$, $X_{2}$ …, $X_{K}$) for the food source (Y)? (2) For outbreaks associated with different etiologies, what food vehicles are predictive of/associated with a specific etiology? For the first question, we will conduct statistical analysis and build multinomial prediction model as explained in the following sections. For the second question, we will use data mining techniques to extract patterns between foods and etiologies. Additionally, to improve our ability to identify the cause of foodborne outbreaks, we will propose a relational analytical approach containing another two aspects: (1) identify the critical foods and the relations between different foods involved in an outbreak caused by specific etiologies, and (2) simulate the food–ingredient relationships to help predict the (ingredient) source of contamination. A food co-occurrence network will be constructed to illustrate the relations between different foods involved in an outbreak, and by analyzing the constructed network, the most important foods causing a specific type of outbreak will be identified. In addition, a food ingredient network describing the relationships between foods and ingredients will be constructed and used with Monte Carlo simulation to predict possible ingredients from foods that cause an outbreak, which could provide insights into the prediction of the possible ingredient sources of contamination when given the name of a food. Details about each of the proposed methods will be illustrated in the following sections.

### 3.3. Data Preprocessing

The data in the raw format are not directly appropriate for data analysis. Therefore, data preprocessing is needed to transform the raw surveillance dataset into a format that can be used for our purposes. Table 1 shows the attribute summary of the original dataset. The focus was on finding patterns in food vehicles (simplified as the vehicle attribute) in outbreaks caused by a confirmed etiology (simplified as the etiology attribute). The vehicle attribute is presented in text format. For text data, basic text preprocessing techniques such as the removal of punctuation, lowercasing, and the removal of useless terms were applied to normalize the food names represented in text format using Python’s *NLTK* 3.0 tool kit. In cases where multiple foods were involved in one outbreak, they were all grouped under this vehicle attribute with punctuation, e.g., comma and semicolon. Basic text preprocessing techniques need to be applied to turn the attributes into a structured format before a data analysis can be conducted [30]. For example, the raw text describing the vehicle attribute was presented as a single string like “beef, meatball; green salad; steak, unspecified”. Rather than having a single string, we used a standard approach to convert it into a set of variables such as “beef”, “green_salad”, and “steak”. Words like “unspecified” were removed from the dataset since those were not informative for identifying the patterns in foods. The etiology attribute, which is presented in nominal format, will be used as an identifier to split the raw dataset into a sub-dataset with outbreaks caused only by the confirmed etiology such as Shiga toxin-producing *Escherichia coli* (STEC). In addition to food vehicle and confirmed etiology, we also investigated other attributes including demographic information (i.e., percentage of cases in an outbreak (in people aged <5, 5–19, 20–49, and ≥50 years), number of cases (number of illnesses reported), multistate outbreak (i.e., cases that occurred in multiple states), exposure setting (where the outbreak occurred, i.e., private or non-private), season (when the outbreak occurred), and outbreak duration (i.e., the number of days between the dates of illness onset and last reported case). The setting attribute (referred to as *location* attribute) is also presented in text format (as shown in Table 1). It will also undergo the same standard preprocessing procedures as those conducted on the *vehicle* attribute. In specific, a location is defined as private when a non-food worker prepares and serves food in that place (e.g., “private home” described for the *location* attribute). All other settings were regarded as non-private. Season was based on onset date of the first case, and it was categorized as spring (March–May), summer (June–August), fall (September–November), and winter (December–February).

### 3.4. Statistical Analysis and Regression Models

Attributes including age, gender, number of illnesses, setting, multistate outbreak, season, and outbreak duration were used as predictors for outbreaks caused by “simple” food vehicles (illustrated in Figure S1). A split-sample method was used to conduct the validation, in which 70% of the dataset was randomly selected as the derivation set and the remaining 30% was used as the validation set. The association between predictor and specific food vehicles was assessed via univariate comparisons using the derivation set. As seen in Table 1, attributes were presented in different formats including nominal (categorical) and numeric (parametric or non-parametric). Thus, different methods were applied. A Kruskal–Wallis test was used to compare the differences in foods for non-parametric predictors, and a Pearson chi-squared ($\chi^{2}$) test was used for categorical predictors [7].

Attributes with univariate significance (*p* < 0.10) and few missing data (<20% missingness) were selected for the prediction model, in which a multinomial logistic regression model was developed to predict the food sources. The model was built based on the derivation set of outbreaks and validated using the remaining outbreak dataset. The performance of the model was evaluated through classification accuracy of both derivation and validation sets. The model predicted the probability of each food source (beef, dairy, and vegetables) such that the predicted probabilities of each food added to 100%. The predicted food was determined by the highest predicted probabilities for each outbreak based on the model. Predicted probabilities were plotted in triangle plots using the Python library, *Plotly (version 3.4.1)*.

### 3.5. Data Mining with Association Rules

To discover the hidden patterns in the relation between the *vehicle* and *etiology* attributes, we used classification models to predict possible pathogens providing the information of food sources (illustrated in Figure S1). The *vehicle* attribute represented in the text format was preprocessed as short documents, in which the foods (words) were transformed to be treated as “tokens”, and each selected etiology was treated as the “label” for building the classification model. Four common etiologies, i.e., *Salmonella enterica* (simplified as *S. enterica*), *Shiga toxin-producing Escherichia coli* (simplified as STEC), *Clostridium perfringens* (simplified as *C. perfringens*), and *Norovirus genogroup I* (simplified as *Norovirus*) and their related outbreaks were chosen as the experimental dataset. Four classification models (support vector machine, naïve bays, logistic regression, and random forest) were used to build the classification models based on the training dataset. A five-fold cross-validation method was used to test the performance of different models, and average accuracy (percentage of correct predictions) was used to select the optimal model.

In addition to the classification method, an association rule was also used to analyze the relations between food vehicles and etiologies [31]. The association rule is another method used to discover correlations between any attributes, but unlike classification, it is unsupervised, meaning that the data are unlabeled [11]. An association rule has three typical measures (*support*, *confidence*, and *lift*) that express the degree of uncertainty about the rule. By definition, *support* is the portion of a pattern occurrence X in the whole dataset, expressed as $support\left(X\right)=P\left(X\right),$ while *confidence* is the probability of finding the pattern occurrence of X∩Y, given the occurrence of X, expressed as $confidence\left(X\to Y\right)=\frac{P(X\cap Y)}{P(X)}$, and *lift* is the ratio of the confidence to the expected confidence, expressed as $lift\left(X\to Y\right)=\frac{confidence(X\to Y)}{P(Y)}=\frac{P(X\cap Y)}{P\left(X\right)P(Y)}$. To discover interesting patterns, a minimum *support* is often used as the first step to filter out frequent patterns whose support is no less than the minimum support. Interesting patterns with higher *confidence* and/or *lift* values were identified as the patterns with stronger associations. In order to implement the two-step process, the *apriori* algorithm will be used.

### 3.6. Network Construction and Centrality Measures

**Figure 1:**
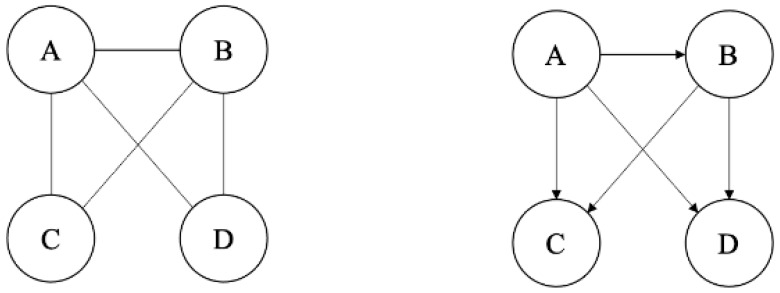
Illustration of undirected (**left**) and directed (**right**) network. (A, B, C, and D are nodes, and the edges connecting the nodes in the undirected network do not have direction with the absence of an arrow, while the edges connecting the nodes in directed network have direction with the presence of an arrow).

After the preprocessing of the vehicle attribute in text format and the removal of non-food terms, outbreaks with less than two food items in their list were excluded. To understand the patterns of food vehicles reported in the same type of contamination, a food co-occurrence network was constructed. The construction, visualization, and analysis of the networks were realized using Python’s package, *NetworkX*. Outbreaks caused by a specific etiology that included two or more food vehicles were used to build the network. Networks are represented by nodes and edges, in which nodes/vertices are entities such as people, and edges/links are connections between the nodes [32]. Edges can be directed, meaning they point from one node to another node, or undirected, as illustrated in Figure 1. The constructed food co-occurrence network was undirected. For each of the unique food items, we defined a **node** as a food item reported in foodborne outbreaks. An **edge** was drawn between nodes if two food items co-occurred in the same outbreak. For example, an outbreak with the vehicle attribute {“beef”, “green_salad”, “steak”} would result in three nodes (beef, green_salad, and steak) and three edges (beef **to** green salad, beef **to** steak, and green_salad **to** steak). Edges have no direction but are weighted, whereas weights represent a simple count of the total number of times any two food items co-occur in a foodborne outbreak. A **co-occurrence**, then, is defined as one discrete edge between nodes with the **weight** of that co-occurrence representing the number of times that co-occurrence was found. Therefore, an edge between beef and green_salad would count as one co-occurrence, and discovering it in 100 outbreaks would have a weight of 100. The food co-occurrence network was analyzed for basic network characteristics using the python package, *NetworkX*. We used Gephi version 0.9.2 for all network visualizations.

The NHANES dataset was used to build a food ingredient network, which described the relationships between foods and ingredients. At the time of download (13 November 2019), the total dataset downloaded was 238 MB. The dataset included multiple files, from which we first created a combined dataset including features of our interest (e.g., food id, food description, ingredient id, and ingredient description) through the FDC_ID and SR_CODE. The names of foods and ingredients were presented in the “description” column and “sr_description” column, which included detailed information of the items other than the general names. For example, the “description” of food (FDC_ID: 336071) was “milk, low sodium, whole”. Secondly, each line was checked by a human annotator to create label names for each food id and ingredient id. All of the detailed descriptions other than the food names were removed by hand. For example, a food name was formatted as “milk”. Foods with similar descriptions were merged as one food name (e.g., whole milk, low-fat milk, and human milk were all labeled as milk), which resulted in 1731 food names in total from 8535 entries. After formatting, the names of all the foods and ingredients were taken as node names for constructing the food ingredient network. The edges were built from the “input_food.xlsx” file based on the connections between them. The weight of the edges was the number of times a food was connected to an ingredient. A directed full food ingredient network was constructed based on the USDA food recipe database. In the food ingredient network, a node represents a food/ingredient item, and a directed link between two nodes (A → B) represents that one node (B) is the ingredient of another node (A), as illustrated in Figure 1.

Centrality measures are several metrics used to determine the importance of different nodes in a network. In the food co-occurrence network, the biggest component, which included the largest number of nodes that were directly or indirectly connected with each other, were extracted for centrality measures. Centrality measures including degree centrality, betweenness centrality, closeness centrality, and eigenvector centrality were used to conduct the network analysis. The centrality terms and their usage in the network analysis are listed below:

- **Degree centrality**: Degree centrality is the simplest centrality measure, defined as the number of other nodes connected with a node (i.e., the number of links that a node has). The degree can be interpreted in terms of the immediate risk of a node for catching whatever is flowing through the network.
- **Betweenness centrality**: Betweenness centrality quantifies the number of times a node acts as a bridge along the shortest path between two other nodes.
- **Closeness centrality**: Closeness centrality is the average length of the shortest paths between the node and all other nodes in the graph. Thus, the more central a node is, the closer it is to all other nodes.
- **Eigenvector centrality**: Eigenvector centrality assigns relative scores to all nodes in the network based on the concept that connections to high-scoring nodes contribute more to the score of the node in question than equal connections to low-scoring nodes.

Finally, degree centrality, the simplest measure, was used to represent the importance/risk of a food associated with one specific etiology based on the food co-occurrence network.

### 3.7. Monte Carlo Simulation Modeling

Through creating the food ingredient network, a hierarchical structure for foods based on ingredients allows us to make maximal use of outbreak data in a consistent fashion to attribute complex foods (foods containing ingredients from different food categories) to specific ingredients. Monte Carlo simulation was performed to predict most common ingredients for food items using Python’s package, *emcee* [33]. Monte Carlo simulation generates results of different ingredients for a given food item on different runs, allowing us to account for the uncertainty of assignments and food placement. After 1000 iterations of running, a frequency distribution of different ingredients predicted for the food item was obtained. By ranking the frequencies, the ingredients with highest frequencies were reported as the simulation results. The simulated results were confirmed with the foods and ingredients reported from historical foodborne outbreaks in the NORS database that were identified as the causes of the outbreaks.

## 4. Results and Discussions

### 4.1. Univariant Analysis between Attributes

From 1998 to 2017, 20,854 outbreaks were recorded in the NORS database. As a sub-set among them, 10,446 (50%) did not identify a food vehicle and were excluded in the analysis. The remaining outbreaks were counted based on the IFSAC category (see Table 2). Since we only focused on “simple” foods, unclassifiable foods, undetermined foods, and multiple foods were excluded, which resulted in 4804 (23%) outbreaks caused by “simple” foods. The top 10 IFSAC category foods associated in the 4804 outbreaks were fish, beef, chicken, mollusks, dairy, pork, fruits, vegetable row crops, turkey, and eggs. The remaining outbreaks, representing either a heterogeneous group of foods or foods with rare outbreak occurrences, were excluded in further analysis. The result of the univariate analysis shows that all identified factors including case demographic and outbreak characteristics (gender, age, number of cases, exposure setting, multistate outbreak, season, and duration) have significant associations with food sources with *p* < 0.01. However, the one-way association analysis only considers all food sources together, whereas the pairwise differences are not shown. Since the null hypothesis for each factor variable was “the mean for different food sources were equal”, a result rejecting the null hypothesis with a *p* value of <0.01 only indicated that “the mean for different food sources was not all equal”, which made it difficult to see the primary factors contributing to the significance. Therefore, we also conducted a test among small groups of food sources. Beef, dairy, and vegetable row crops (simplified as vegetables) contributing to major multistate outbreaks and causing a high number of illnesses were chosen as food sources to better demonstrate the associations between the identified factors and the food sources.

As shown in Table 3, gender and age distribution differed among three food sources (beef, dairy, and vegetables), with outbreaks caused by vegetable raw crops having the highest median percentage for females, whereas there was no significant gender difference in beef and dairy outbreaks. Similar results were found in the study of Shiga toxin-producing *Escherichia coli* (STEC) outbreaks [7]. This might be attributed to the fact that the consumption of fruits and vegetables were significantly higher among women than men. Dairy outbreaks had the highest median percentage of children and adolescents, and the lowest median percentage of the elderly, which might also be associated with people’s food consumption habits. Beef and dairy outbreaks had significantly smaller numbers of illnesses than those caused by vegetables. On the other hand, the proportion of multistate outbreaks was significantly higher in vegetable outbreaks than in dairy or beef outbreaks. Since multistate outbreaks usually involve a large number of illnesses, it is natural to understand why vegetable outbreaks were associated with more cases. In addition, vegetable outbreaks had a higher proportion in non-private exposure settings (e.g., restaurants) than beef or dairy outbreaks, which can also be correlated to the proportion of multistate outbreaks and number of cases. Though a significant difference (*p* < 0.01) was found when analyzing the association between season and ten food sources, no significant association was identified in beef, dairy, and vegetable outbreaks (*p* = 0.10). In the study of STEC outbreaks, the researchers identified that season was a significant predictive factor of food sources. They found that STEC outbreaks caused by vegetables occurred mostly in the fall, whereas STEC outbreaks caused by beef mostly occurred in the summer [7]. In this study, we did not classify datasets based on etiologies and found that summer was the primary season for beef, dairy, and vegetable outbreaks, which might be due to food consumption factors such as availability, cost, and seasonal events. Lastly, a significant association was found between outbreak duration time (days) and food sources, with dairy outbreaks having the longest median percentage of duration time. This might be attributed to the longer incubation time of the primary etiology (e.g., *Listeria monocytogenes*: 2–10 weeks) causing dairy outbreaks than that of beef or vegetable outbreaks (e.g., *Escherichia coli*: 1–10 days). Attributes with univariate significance (*p* < 0.01) and few missing data (<20% missingness) were selected as independent variables to build the prediction models.

### 4.2. Multinomial Regression for Predicting Food Sources

**Figure 2:**
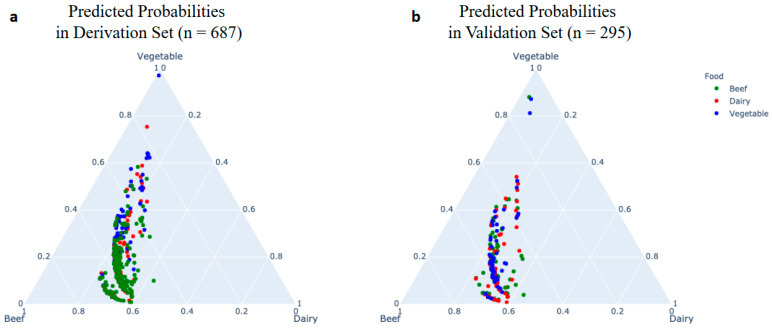
Predicted probability of beef, dairy, and vegetable food sources as a function of actual food sources. The three-way predicted probabilities of beef, dairy, and vegetable food sources in the derivation set (**a**) and the validation set (**b**) such that the total predicted probability for each outbreak sums to 100%.

Four selected factors (percentage female, number of cases, multistate outbreak, and exposure setting) were used as independent variables. To predict more than two categorical placements (beef, dairy, and vegetables) as the dependent variables based on multiple independent variables either in dichotomous format (multistate outbreak and exposure setting) or in continuous format (percentage female and number of cases), a common method is multinomial logistic regression. The total size of the three outbreaks after removing the ones with missing values was 982, of which 687 (70%) outbreaks were randomly selected as the derivation set to build the prediction model, and the remaining 295 outbreaks were used as the validation set. The performance of the model for predicting food sources among beef, dairy, and vegetables is shown in Figure 2 through three-way predicted probabilities. The average predicted probability in the derivation set was 0.57 ± 0.06 (mean ± standard deviation) for beef outbreaks, 0.28 ± 0.06 for dairy outbreaks, and 0.28 ± 0.16 for vegetable outbreaks. The average predicted probability in the validation set was 0.57 ± 0.08 for beef outbreaks, 0.28 ± 0.06 for dairy outbreaks, and 0.23 ± 0.17 for vegetable outbreaks. The model chose the food with highest probability as the predicted food. Compared with the actual food sources, the model correctly classified 360 out of 373 beef outbreaks, 0 out of 189 dairy outbreaks, and 23 out of 121 vegetable outbreaks in the derivation set. The predicted probabilities for the beef outbreaks clustered near the “beef” apex of the plot in Figure 2, and only a few outbreaks predicted as “vegetable” clustered near the “vegetable” apex. Noticeably, there were no outbreaks clustered near the “dairy” apex, dispersing between the “beef” and “vegetable” apexes. This indicates that the model only performs well for predicting beef outbreaks and not for vegetable or dairy outbreaks. Similarly, the model correctly classified 151 of 157 beef outbreaks, 0 of 86 dairy outbreaks, and 8 out of 52 vegetable outbreaks in the validation set. According to the univariate analysis results in Table 3, no significant differences were found between beef and dairy for the four selected predictors (percent female, number of cases, exposure setting, and multistate outbreak in terms of proportion). Therefore, it is unsurprising to see that the model misclassified most “dairy” outbreaks as “beef” outbreaks. On the other hand, the biased sample distribution was another reason for the inferior performance of the prediction model.

### 4.3. Classification Models for Predicting Etiology Based on Food Vehicles

**Figure 3:**
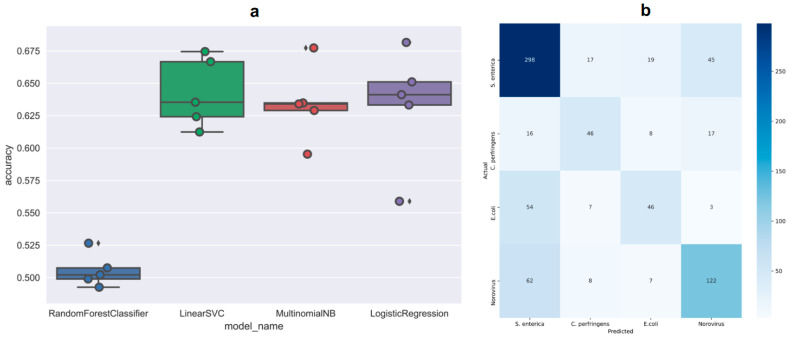
Performance of different machine learning models in classifying the four etiologies. Average accuracies of four classification models with five-fold cross-validation (**a**); confusion matrix of the optimal classification model (**b**).

The average accuracy of each model for predicting the correct etiology was calculated, and the results were plotted based on five-fold cross-validation (see Figure 3a). The support vector machine model was selected as the best classification model for its highest average accuracy (0.64 ± 0.05) than the other three models. Based on the model, we calculated the performance matrices for each etiology. As shown in Table S1, a total number of 775 data points with labels was used to test the performance of the model, with 379 outbreaks caused by *S. enterica*, 87 outbreaks caused by STEC, 110 outbreaks caused by *C. perfringens*, and 199 outbreaks caused by *Norovirus*. The model predicted best for *S. enterica* and worst for *C. perfringens*, with the other two in the order of *Norovirus* > STEC. A confusion matrix is commonly used to visualize the performance of a classification model/classifier on a set of test data for which the true values are known. The confusion matrix of the trained support vector machine classifier more clearly shows the prediction results for each etiology (see Figure 3b), where the x-axis is the predicted etiology, and the y-axis is the actual etiology. The diagonal numbers represent the correct predictions for each etiology in which the darker the color the better the performance. It can be clearly seen that the model performed best for *S. enterica*, followed by *Norovirus*, *C. perfringens*, and STEC. Also, high proportions of STEC outbreaks and *Norovirus* outbreaks were wrongly predicted as *S. enterica* outbreaks, while the largest proportion of incorrect predictions for *S. enterica* was *Norovirus*.

One reason for the differences in model performance among the different etiologies could be the sample bias or imbalanced classes. For instance, there are 339 instances of STEC outbreaks in the database out of a total of 3092 instances, while the number of *S. enterica* outbreaks is 1378. The problem of this imbalanced dataset is that a model will ignore the minority class value. Resampling (e.g., sample with replacement) is a common method often used to address this type of problem by giving higher chances of being sampled to the minority classes. For example, Thakur et al. (2010) [11] conducted resampling when building their binary classification models. However, as mentioned by the authors, resampling introduces bias by overemphasizing some of the minority classes, and the original distribution pattern of the outbreak types would also be changed in the process. We also tried the resampling method in our study, and found that the overall prediction accuracy, especially for the minority classes, was increased. Since the objective of this study was to develop classification models that can be potentially used to predict etiologies from food vehicles in real-life outbreaks, keeping the original pattern was of significance in the data analysis process. Thus, a resampling process was not reported in the study. Differently to the previous study, ours was a multiclass problem in which we tried to discriminate all etiologies of interest simultaneously [11].

### 4.4. Association Rule Mining between Food Vehicles and Etiologies

Unlike classification, association rule mining is an unsupervised learning method that will find all the relationships/association rules between any attributes [11]. In this study, we expected to see the patterns that could provide insights into what types of etiologies were caused by what types of food vehicles. Hence, only the association rules related to the etiologies of interest were included in the result. As shown in Table 4, by setting the minimum support to 0.005, we obtained all of the association rules (X→Y, where X is a food vehicle and Y is an etiology) for each etiology ranked by their *lift* values. Only strong rules with *lift* values higher than 1.0 were included in the result. It was observed that the *lift* value of roast beef, in which *C. perfringens* was involved, is 5.35, meaning that the probability that roast beef will be involved in a *C. perfringens* outbreak is 5.35 times higher than the general probability of roast beef in the whole dataset. Similar interpretations can be made for other rules identified with high *lift* values. For STEC, the three association rules indicated that the consumption of beef or lettuce was strongly linked to STEC outbreaks. For *norovirus* and *S. enterica*, many rules were identified, and the food vehicles linked with them were more diverse than the ones involved in the *C. perfringens* outbreaks or STEC outbreaks. From the classification results, it was found that most false predictions of *norovirus* outbreaks were classified as *S. enterica* outbreaks. The diverse distribution of food sources might be one reason, as the same food vehicles might be highly linked to multiple etiologies. For example, cheese was both associated with *Norovirus* (*confidence*: 0.32; *lift*: 1.22) and *S. enterica* (*confidence*: 0.61; *lift*: 1.26) based on Table 4.

### 4.5. Characteristics of the Food Co-Occurrence Network

**Figure 4:**
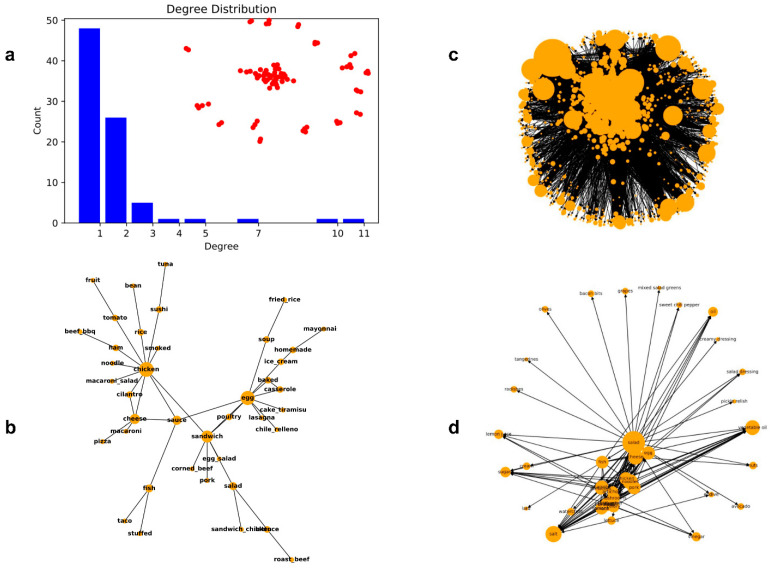
Network construction and visualization. Degree distribution of food co-occurrence network for *S. enterica* outbreaks (**a**); visualization of the largest component of the food co-occurrence network for *S. enterica* outbreaks (**b**); visualization of the full food ingredient network (**c**); and ego network of the “salad” node from the full network (**d**).

Multiple foods may be involved in one outbreak, in which if the co-occurrence of different foods in the outbreaks caused by a specific etiology is high, it might indicate a strong inner relationship between the foods. Through constructing a food co-occurrence network, the roles played by different foods in a specific outbreak can be easily seen. In addition, centrality measures could also provide informative data to identify the most important nodes/foods that play roles in that type of outbreak. For example, the *degree centrality* (simplified as degree) was calculated for each node in the network of *S. enterica* outbreaks. The degree value was calculated based on the number of other nodes connected to one node. The degree distribution shows how many foods share the same degree value. Figure 4a shows the degree distribution (the blue bar) of the food co-occurrence network for *S. Enterica* outbreaks, and the dispersion of all nodes (the red dots) involved in that network. The biggest component in the center consists of many red nodes/foods that are highly connected to each other, while many small components that are dispersed around consist of very few foods that are weakly connected to each other. The degree distribution also shows the same phenomenon with many foods only connecting to one other food (degree value = 1), one food connecting to eleven other foods (degree value = 11), and many other foods with very high degree values such as 5, 7, and 10. Based on the definition of *degree centrality*, the one with high degree value is regarded as the important food involved in outbreaks. By visualizing the biggest component, Figure 4b shows the links between different foods, in which the size of a node is proportional to the degree value. Thus, it is easily seen that chicken, the largest node, is the one playing the most important role in this network, and other foods with large node sizes such as eggs, sandwiches, cheeses, and sauces are also critical foods involved in *S. enterica* outbreaks. In addition, the placement of different foods in the graph also provides a clear idea of the centered and bordered foods. However, there are some limitations of the text processing methods. For example, some non-food items, such as “homemade” as an adjective of a food, are also recognized as food items and included in the network, though this will not affect the analysis of the roles played by the food items.

The definition of the most important nodes differs with different centrality measures. Therefore, we also calculated the values for the other three common centrality measures including *betweenness centrality*, *closeness centrality*, and *eigenvector centrality* for the four selected types of outbreaks. Table S2 shows the top 10 food items identified for each type of outbreak based on different centrality measures. The food items are almost the same using different centrality measures, except the rankings vary. For example, chicken, sandwiches, and eggs are the top three food items accounting for *S. enterica* outbreaks, while their ranks are different based on different centrality measures. When using degree, betweenness, or eigenvector as the measure for centrality, chicken is the most important item, followed by egg and sandwich. When using closeness, sandwich is the most important item, followed by chicken and egg. The variation in the results is attributed to the different definitions and formulas for calculating the metrics based on the network structure. In terms of the relevance to foodborne outbreak investigation, the degree centrality, which is the number of connections that one food has to other foods, is more important than the other metrics. And the most important food items are either raw foods/ingredients (e.g., chicken and egg) or complex foods (e.g., sandwich) that include multiple ingredients.

Cross-contamination, defined as a general term that refers to the transfer, direct or indirect, of a bacteria or virus from a contaminated product to a non-contaminated product, is associated with a high profile of foodborne outbreaks [34]. Deficient hygiene practices, contaminated equipment, contamination via food handlers, processing, or inadequate storage are typical factors in cross-contamination events [35]. Also, cross-contamination can occur during food consumption [11]. The discovery of frequently occurring patterns where multiple foods caused a foodborne outbreak through food co-occurrence network provides knowledge that is useful for designing safe food handling practices for food handlers and consumers. For example, in *S. enterica* outbreaks, ingredient foods such as chicken and eggs may impose potential cross-contamination risks on prepared foods such as sandwiches if they are contaminated with *S. enterica* and not properly handled during food preparation and consumption.

### 4.6. Characteristics of the Food Ingredient Network

The visualization of the full food ingredient network is shown in Figure 4c, in which node A pointing to node B means B is A’s ingredient, and the size of a node is proportional to its degree centrality or the number of connections the node linked with. There are 1731 nodes including food names and ingredient names in the full network. An ego network of the “salad” node was selected from the full network to show the relationships between foods and ingredients (Figure 4d). There are many ingredients that “salad” points to, and some ingredients have their sub-ingredients. For example, there are chain relationships like “salad” → “vegetable” → “lettuce”. The weights of the edges in the food ingredient network were calculated based on the prevalence of an ingredient (node B, e.g., lettuce) in the recipe of a particular food (node A, e.g., salad) if A points to B. A Monte Carlo simulation was applied on the constructed food ingredient network to identify the ingredient source of contamination for foods, particularly complex foods consisting of multiple ingredients (e.g., salad), involved in foodborne outbreaks. The intuition behind the simulation is like a random walk on the food ingredient network. The higher the weight of an edge from node A to node B, the higher the probability that B will be visited and returned as the result in one simulation round. By running the Monte Carlo simulation for 10,000 times, a list of ingredients with the highest probabilities were obtained. Table S3 lists the most prevalent ingredients of some “complex” foods, including “salad”, “sandwich”, “pizza”, “taco”, “pasta”, and “lasagna” from the simulation results. It is surprising to see that some ingredients that rarely appeared in typical foods, such as “parsley” in “salad” and “grapes” in “sandwich”, turned out to be some of the most prevalent ingredients. Also, essential ingredients, such as “salt”, “oil”, and “fat”, are prevalent in all of the example foods. As the weight of a connection between two nodes was defined as the number of times that an ingredient appeared in the recipe of a food, the essential ingredients that were included in almost all recipes would stand out as the dominant ingredients. However, the other dominant ingredients such as “parsley” and “graphs” might be attributed to the significant number of vegan recipes with these ingredients. Based on the data reported by historical foodborne outbreaks, the top contaminated ingredients of selected “complex” foods are listed in Table S4, which was used as a reference to evaluate the results from the Monte Carlo simulation. The most common contaminated ingredients such as “leafy green” in “salad” and “sandwich” are not present in the simulation results, which is attributed to the term variations in the two datasets. The ingredient terms in the recipe data are more specific than those from the outbreak data. And many contaminated ingredients in outbreaks such as “egg” in “pasta” and “lasagna” are not prevalent in the simulation results, indicating no strong connections between the prevalence of the ingredients in food recipes and the occurrence of contaminated ingredients reported in historical foodborne outbreaks. In this case, the food ingredient network was updated by re-assigning the weight of links between two nodes, which we defined as the number of times that an ingredient was identified as the contaminated source for a food vehicle in a historical outbreak. If a pre-existing node B is not present as a contaminated ingredient for a node A, then the weight would be assigned as zero. To note that, the connections between the two nodes were not removed; in other words, the structure of the food ingredient network remained the same. After running the Monte Carlo simulation for 1000 times on the network with updated weights, the results directly reflected the prevalence of popular contaminated ingredients for the selected “complex” foods, except for some ingredients (e.g., “leafy green”) that failed to be normalized across the two datasets.

A network analysis has been widely used in food and ingredient analysis for the publicly available online recipes [36]. The major purpose of the studies was focused on identifying the patterns in ingredient combinations, or in other words, the flavor-pairing patterns in different cuisines [17]. A few recent studies explored the relationships between foods and ingredients for a better understanding of food intake behaviors in the United States with a network analysis [22]. This work is the first time that a network was introduced to the analysis of foods and ingredients involved in foodborne outbreaks. By constructing the food ingredient network through recipe data and a Monte Carlo simulation, we could obtain a list of ingredients ranked by the prevalence in the recipe of a given food. However, the results were more consistent with the data from historical outbreaks when the weights of the edges were re-assigned as the occurrence of a node identified as a contaminated ingredient for another node. Normalization is another challenge in comparing results from different datasets. The results are also limited by using only the NHANES dataset to construct the food ingredient network, which includes a significant number of vegan recipes. The structure of the food ingredient network would be improved if a more comprehensive recipe dataset is available. The major contribution of this paper is that the food ingredient network coupled with the Monte Carlo simulation provides a new approach for attributing ingredient sources of contamination for “complex” foods. By improving the food ingredient network so that it can better represent the food composition and weighting of each ingredient considering both its risk (occurrence as a contaminated ingredient in historical foodborne outbreaks) and its prevalence (occurrence as an ingredient in the recipe of a food), this approach would have the potential to provide the most probable ingredients of contamination when given a complex food involved in a future outbreak.

## 5. Conclusions

In this study, we investigated the associations between outbreak characteristics and food vehicles. Interesting factors (percentage female, number of cases, multistate outbreak, and exposure setting) with significant associations were selected to build the model to predict food sources, in which beef, dairy, and vegetables were the targeted “simple” food sources. From the result of the multinomial regression model, we observed that the model predicted well for beef outbreaks, while it did not perform well for the other outbreaks, especially the dairy outbreaks. The imbalanced sample size for the different food sources might be one of the major factors in the misclassification. This study provides evidence for the feasibility of using prior cases and outbreak characteristics to predict “simple” food sources in foodborne outbreak investigations. The relations between food vehicles and the etiologies involved in outbreaks were also investigated. Through the data mining of food vehicles in free-form text and multi-class classification, we illustrated the performances of different models in predicting etiologies of interest. The support vector machine classifier was identified as the optimal model. Similarly, the results that the model performed best for *S. enterica* outbreaks and most misclassifications were wrongly predicted as *S. enterica* outbreaks might also be attributed to the sample bias raised from the imbalanced data size. Incomplete data is another factor accounting for the misclassifications. For example, the age attribute, which was of significance in the univariate analysis, was not included in the prediction model due to the incomplete data. Thus, the performance would be improved if larger datasets and more complete data are available. Apart from classification, the association rule mining provided insights of what food vehicles were highly linked with outbreaks involved with specific etiologies. When multiple foods were involved in one outbreak, we identified the most important foods by constructing the food co-occurrence network and a centrality analysis. In addition, the food co-occurrence network could provide insights of cross-contamination for outbreaks caused by specific etiologies. When complex foods consisted of multiple ingredients are involved in an outbreak, a Monte Carlo simulation of the food ingredient network from the recipe data could provide ideas of prevalent ingredients. The results were evaluated with the historical outbreak data, which showed that the weights defined as the occurrence of an ingredient in a food recipe did not reflect their chances of being detected as contaminated sources in outbreaks. However, the methodology provides a way to gain first-step insights into the most common ingredients of a “complex” food in an automatic manner. By updating the weights of the links between two nodes, the simulation results were improved. Incorporating more comprehensive recipe data into the network construction would improve the food ingredient network structure and the simulation results.

The complexity of the global food industry and modern challenges to food safety show that the outbreak investigation needs to be more efficient. The data-mining approach described in this study may provide insights for health authorities in improving the understanding of critical attributes contributing to the food source of contamination and assisting their hypothesis-generating process. The links between food sources and etiologies could also be used to assist food manufactures in their risk assessment by developing timely and corrective food safety measures for specific types of contaminations, so that products can not only be safe, but also have a longer commercial life.

## Figures and Tables

**Table 1:** Attribute summary of the original dataset.

Attribute	Type	Description
Etiology	Nominal	Cause of outbreak, e.g., *Salmonella enterica*
State	Nominal	State where the outbreak occurred
Month	Nominal	Month when the outbreak occurred
Illnesses	Numeric	Number of illnesses reported
Hospitalizations	Numeric	Number of hospitalizations reported
Deaths	Numeric	Number of deaths reported
Food Vehicle	Text	Food item/s that caused the outbreak
Setting	Text	Location where food was consumed, e.g., restaurant

**Table 2:** IFSAC category of outbreaks with identified food vehicles.

IFSAC Category	Number of Outbreaks
Multiple	4045
Undetermined	1126
Fish	892
Beef	678
Chicken	501
Unclassifiable	433
Mollusks	339
Dairy	324
Pork	318
Fruits	245
Vegetable Row Crops	215
Turkey	190
Eggs	186
Other	182
Grains-Beans	178
Seeded Vegetables	136
Crustaceans	109
Root/Underground	79
Sprouts	53
Fungi	40
Herbs	33
Game	30
Nuts-Seeds	26
Other Meat	16
Oils-Sugars	12
Other Poultry	11
Other Aquatic Animals	11

**Table 3:** Univariate analysis between identified factors and food sources.

Identified Factors	Beef	Dairy	Vegetable	*p*-Value
Number of outbreaks	678	324	215	
Total cases	11,649	6979	9910	
Gender ^a^				
Male	50 (33, 60)	50 (38, 61)	37 (25, 50)	<0.01
Female	50 (40, 66)	50 (36, 60)	62 (50, 75)	<0.01
Missing ^b^	121 (18)	26 (8)	27 (13)	<0.01
Age (years) ^a^				
% < 5	0 (0, 0)	0 (0, 11)	0 (0, 0)	<0.01
% 5–19	0 (0, 14)	0 (0, 22)	0 (0, 11)	<0.01
% 20–49	33 (12, 57)	34 (10, 50)	43 (21, 50)	0.37
% >= 50	0 (0, 24)	0 (0, 12)	8 (0, 29)	<0.01
% Unknown	0 (0, 5)	0 (0, 0)	0 (0, 4)	0.42
Missing ^b^	556 (82)	158 (49)	126 (59)	<0.01
Number of cases ^a^	6 (3, 20)	7 (3, 15)	16 (9, 30)	<0.01
Exposure setting ^b^				<0.01
Private	125 (18)	53 (16)	18 (8)	
Non-private	533 (79)	252 (78)	185 (86)	
Missing	20 (3)	19 (6)	12 (6)	
Multistate Outbreak ^b^	50 (7)	24 (7)	45 (21)	<0.01
Season ^b^				0.1
Spring	154 (23)	62 (19)	43 (20)	
Summer	182 (27)	103 (32)	73 (34)	
Fall	204 (30)	88 (27)	44 (20)	
Winter	204 (30)	71 (22)	55 (26)	
Duration (days) ^a^	3 (2, 14)	11 (4, 36)	6 (3, 16)	<0.01
Missing ^b^	437 (64)	91 (28)	84 (39)	<0.01

-a: data presented as median (IRQ), *p*-value from Kruskal-Wallis. -b: data presented as proportion (%), *p*-value from chi-square.

**Table 4:** Association rules found for outbreaks caused by specific etiologies.

Etiology	Food Vehicle	Confidence	Lift
*C. perfringens*	Roast beef	0.69	5.35
	Gravy	0.56	4.37
	Beef	0.33	2.55
	Turkey	0.36	2.76
	Chicken	0.21	1.63
STEC	Ground beef	0.60	4.80
	Beef	0.27	2.15
	Lettuce	0.26	2.05
	Ice	0.94	3.55
	Raw oyster	0.88	3.34
	Sandwich	0.86	3.25
	Green salad	0.75	2.85
*Norovirus*	Salad	0.74	2.81
	Fruit	0.70	2.64
	Lettuce	0.62	2.38
	Coleslaw	0.59	2.24
	Cake	0.56	2.11
	Cheese	0.32	1.22
*S. enterica*	Egg	0.98	2.02
	Cantaloupe	0.81	1.68
	Fish	0.80	1.66
	Ice cream	0.79	1.64
	Alfalfa sprout	0.73	1.51
	Tomato	0.71	1.47
	Sushi	0.71	1.46
	Chicken	0.69	1.43
	Pork	0.63	1.30
	Cheese	0.61	1.26
	Salsa	0.60	1.24
	Turkey	0.53	1.10

## Data Availability

The datasets collected and analyzed during the current study are available from the corresponding authors upon reasonable request. The CDC dataset of historical foodborne outbreaks are available through the NORS data request application by emailing the NORS dashboard mailbox.

## Supplementary Materials

The following supporting information can be downloaded at https://www.mdpi.com/article/10.3390/foods12203825/s1, Figure S1. Schematic structure of the experimental design. Figure S2. Basic statistics of the NORS dataset (left: number of foodborne outbreaks with identified or unidentified food vehicles; right: composition of the outbreaks with identified food vehicles). Table S1. Performance matrices of classification model in predicting for four etiologies. Table S2. Top 10 food items based on different centrality measures. Table S3. Most common ingredients of “complex” foods from Monte Carlo simulation. Table S4. Contaminated ingredients associated with “complex” foods in historical outbreaks.
