# Supplementary material for: Epidemiological Data Mining for Assisting with Foodborne Outbreak Investigation
Source: Foods. 2023 Oct 19;12(20):3825. doi: 10.3390/foods12203825 (PMC10606626; doi:10.3390/foods12203825)
Supplement: Supplementary file 1 [file foods-12-03825-s001.zip › foods-2664043-supplementary.pdf]

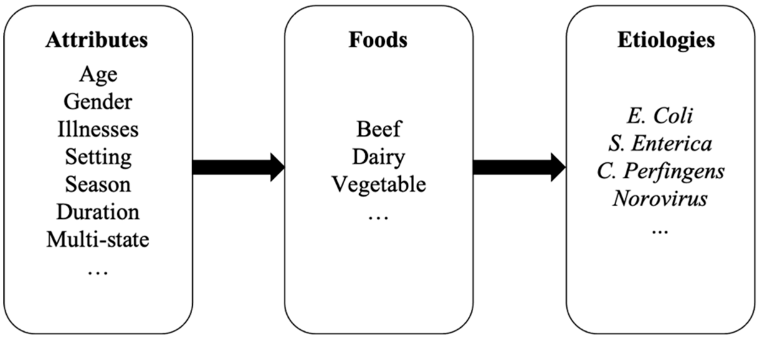

Figure S1. Schematic structure of the experiment design

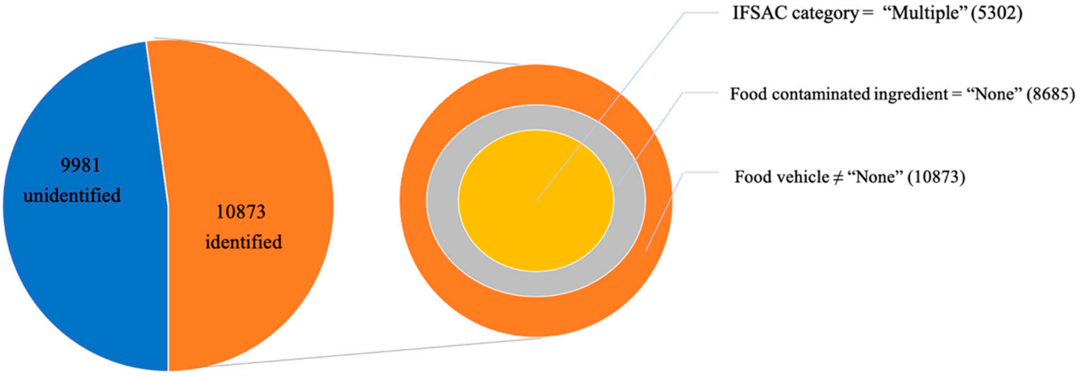

Figure S2. Basic statistics of the NORS dataset (left: number of foodborne outbreaks with identified or unidentified food vehicles; right: composition of the outbreaks with identified food vehicles.)

Table S1. Performance matrices of Classification model in predicting for four etiologies

**Etiologies**	**precision**	**recall**	**f1-score**	**support**
Salmonella enterica	0.69	0.79	0.74	379
Shiga toxin-producing Escherichia coli	0.59	0.53	0.56	87
Clostridium perfringens	0.57	0.42	0.48	110
Norovirus genogroup I	0.65	0.61	0.63	199
Average / Total	0.65	0.66	0.65	775

Table S2. Top 10 food items based on different centrality measures

Salmonella enterica	*Norovirus genogroup I*	*Clostridium perfringens*	*Escherichia coli, Shiga toxin-producing*
Top 10 food items of the highest degree centrality			
chicken	salad	gravy	ground_beef
egg	lettuce	chicken	bean
sandwich	sandwich	roast_beef	salad
cheese	vegetable	ground_beef	pork
sauce	cheese	noodle	rice
pie	ground_beef	rice	vegetable
fish	ice	enchilada	meatball
pork_bbq	bread	taco	cheeseburger
salad	pineapple	turkey	parsley
beef	celery	mashed_potato	house_salad
Top 10 food items of the highest betweenness centrality			
chicken	salad	gravy	ground_beef
egg	lettuce	roast_beef	bean
sandwich	tortilla	chicken	salad
sauce	sandwich	ground_beef	vegetable
salad	cheese	noodle	meatball
cheese	vegetable	rice	cheeseburger
fish	bread	enchilada	pork
ice_cream	potato_salad	taco	parsley
homemade	ground_beef	turkey	house_salad
rice	ice	mashed_potato	dip
Top 10 food items of the highest closeness centrality			
sandwich	salad	gravy	ground_beef
chicken	lettuce	roast_beef	bean
egg	sandwich	turkey	meatball
sauce	bread	mashed_potato	cheeseburger
cheese	pasta	sausage	hamburger
poultry	vegetable	gravy_beef	steak
salad	tortilla	chicken	pork
cilantro	potato_salad	ground_beef	rice
ham	fruit	shredded	salad
tomato	punch	meatball	dip
Top 10 food items of the highest eigenvector centrality			
chicken	salad	gravy	vegetable
egg	lettuce	roast_beef	salad
sandwich	sandwich	turkey	meatball
sauce	bread	mashed_potato	ground_beef
cheese	vegetable	sausage	cheeseburger
cilantro	pasta	gravy_beef	bean
poultry	punch	chicken	pork

tomato	fruit	ground_beef	parsley
ham	potato_salad	shredded	house_salad
rice	seafood	meatball	dip

Table S3. Most common ingredients of “complex” foods from Monte Carlo simulation

**Salad**	**Sandwich**	**Pizza**	**Taco**	**Pasta**	**Lasagna**
Parsley	Salt	Pineapple	Olives	Cream	Salt
Olives	Olives	Salt	Cream	Salt	Cream
Salt	Grapes	Cheese	Garlic	Garlic	Garlic
Sauerkraut	Cream	Peppers	Salt	Milk	Milk
Fat	Cheese	Chicken	Cheese	Cheese	Cheese
Cheese	Vinegar	Onions	Vinegar	Fat	Fat
Lettuce	Fat	Oil	Lettuce	Crackers	Margarine-like
Margarine-like	Lettuce	Squash	Lime juice	Margarine-like	spread
spread	Watercress	Mushrooms	Oil	spread	Peppers
Dressing	Lemon juice	Fat	Peppers	Chicken	Oil
Lemon juice	Chicken	Juice	Chicken	Oil	Onions
Pork	Oil	Pickle	Onions	Corn	Celery
Oil	Onions	Pimento	Fat	Onions	Spinach
Chicken	Fish	Pork	Fish	Carrots	Carrots
Salami	Celery	Sweet chili	Egg	Broccoli	Broccoli
Fish	Spinach	pepper	Juice	Dressing	Mushrooms
Cucumber	Cucumber	Sugars	Lime	Egg	Eggplant
Onions	Carrots	Milk	Rice	Beef	Egg
Turkey	Radish	Vegetable oil	Dressing	Margarine	Squash
Celery	Beef	Bread	Tortillas	Pork	Dressing
Tomato		Sausage		Tomato	Beef

Table S4. Contaminated ingredients associated with "complex" foods in historical outbreaks

Salad	Sandwich	Pizza	Taco	Pasta	Lasagna
Leafy green	Leafy green	Pork	Beef	Egg	Egg
Tomato	Chicken	Cheese	Chicken	Vine-stalk	Beef
Chicken	Lettuce	Chicken	Fish	Tomato	
Lettuce	Bread	Mushrooms	Legume	Dressings	
Egg	Tomato	Cream	Mahi	Potato	
Potato	Beef	Bread	Pork	Root	
Root	Turkey	Fruit	Egg	Sauces	
Vine-stalk	Pork	Lettuce	Fungus	Shrimp	
Dressings	Cheese	Dressings	Leafy green	Beans	
Cheese	Vine-stalk	Garlic	Lettuce	Chicken	
Sauces	Egg	Broccoli	Pork	Leafy green	
Fruit	Fish	Beef	Spices	Mussels	
Pork	Fin	Basil	Tortillas		
Fish	Fruit	Potato	Wraps		
Onion	Ham	Root	Wahoo		
Cucumber	Potato	Salt			
Shellfish	Mayonnaise	Sausage			
Turkey	Basil	Sourdough			
Tuna	Root	Sugar			
Beef	Onion	Sauces			
Water	Mushroom	Tomato			
Carrots	Deli meat				
Celery	Peppers				
Pineapple	Pickles				
Grapes	Sprouts				
Cream					
Basil					
Vinegar					
Melon					
